# Safety and Cost-Analysis of Same-Day Versus Next-Day Discharge Following Complex Ablation Procedures: A Single-Centre Observational Study

**DOI:** 10.3390/jcdd13100501

**Published:** 2026-10-04

**Authors:** Mònica Jurado, Carlos-Eduardo González-Matos, Benjamin Casteigt, Jesús Jiménez, Pasquale Falzone, Ermengol Vallès

**Affiliations:** 1 Faculty of Medicine, Universitat Pompeu Fabra, 08003 Barcelona, Spain; monicajuradoramal@gmail.com (M.J.); jjimenezlopez@hmar.cat (J.J.); 2 Department of Cardiology, Electrophysiology, Hospital del Mar, 08003 Barcelona, Spain; cgonzalezmatos@hmar.cat (C.-E.G.-M.); bcasteigt@hmar.cat (B.C.); pvfalzone@hmar.cat (P.F.)

**Keywords:** catheter ablation, safety, cost-analysis, complex ablation, same-day discharge

## Abstract

(1) Introduction and objectives: The most common practice after a complex ablation procedure (CAP) is next-day discharge (NDD) following an appropriate observation period. However, the use of same-day discharge (SDD) has increased substantially since the COVID-19 pandemic. We aimed to evaluate the safety and cost-analysis of an SDD strategy for patients undergoing CAP. (2) Methods: This was a single-centre, retrospective observational study of patients undergoing CAP between January 2021 and July 2024, with intracardiac echocardiography used in all procedures. (3) Results: A total of 250 CAPs were performed, with a complication rate of 20.4% in the initial cohort. Forty-five patients were excluded, mainly because of significant procedural complications or severe pre-ablation medical conditions. The final analysis included 205 patients: 70 in the SDD group and 135 in the NDD group. Multiple patient and procedural characteristics were evaluated and compared between the groups. Among patients included in the final analysis, the overall complication rate was low (8.8%), with no differences between groups (early minor complications, 5.4%: 8.6% vs. 3.7% for SDD vs. NDD, *p* = 0.191; late major complications, 0.5%: 0% vs. 0.7%, *p* = 1; late minor complications, 3.4%: 2.9% vs. 3.7%, *p* = 1). There were five deaths (2.4%), none of which were procedure related. The SDD policy for CAP during this period would have saved €479.84 ± €248.09 per patient. (4) Conclusions: An SDD strategy was feasible and cost-saving and seemed to not increase periprocedural complications compared with an NDD strategy.

## 1. Introduction

In 2023, a total of 26,207 ablation procedures (APs) were performed in Spain [1]. The increase in the indications and technological advances have enabled increasingly complex ablation procedures (CAPs), such as those requiring transseptal puncture [1]. Historically, these procedures have been performed during a hospital admission, imposing substantial costs [2] on the healthcare system. The COVID-19 pandemic limited access to health services worldwide [3,4], leading to a marked reduction in hospital admissions for conditions other than COVID-19. This reduction was partially offset by a substantial increase in early-discharge procedures [5], including complex ablations. Protocols involving shorter hospital stays were rapidly established worldwide to maintain activity. As part of this response, our centre introduced a “same-day discharge” (SDD) strategy as an alternative to the standard-of-care “next-day discharge” (NDD) strategy. Because of its benefits in terms of costs and bed availability, this strategy was continued after the pandemic. The present study aimed to evaluate the safety and cost-analysis of SDD in patients undergoing complex ablation.

## 2. Methods

### 2.1. Study Design

This was a single-centre, retrospective, longitudinal observational study evaluating the safety and cost-analysis of an SDD strategy compared with NDD after CAP, with intracardiac echocardiography (ICE) used in all procedures. The study was approved by the hospital ethics committee (registry number 2025/12021) in accordance with the Declaration of Helsinki. The committee waived the requirement for informed consent because of the retrospective design and anonymisation of the study population.

### 2.2. Study Population and Definition of CAP

Patients who underwent CAP between January 2021 and July 2024 were retrospectively identified. CAP was defined as any AP requiring a left-sided approach, either through transseptal puncture or retroaortic arterial access. To be eligible for analysis, patients were required to meet all inclusion criteria and none of the exclusion criteria.

### 2.3. Inclusion Criteria

Age between 18 and 80 years;

Patients who underwent a CAP.

#### 2.3.1. Exclusion Criteria

Patients without data in the shared medical electronic records.

Patients not able to be followed by our centre after the CAP.

Patients who refused same-day hospital discharge and therefore could not be allocated according to either strategy.

Patients admitted because of severe conditions who did not meet the criteria for discharge after the procedure.

#### 2.3.2. Criterion Precluding SDD Strategy (Exlusion Criterion)

Patients who experienced a significant complication during CAP or the subsequent 8 h postprocedural monitoring period. In such cases, hospital admission was mandatory for patient safety, and any other necessary measures were undertaken.

Patients were subsequently categorised into two groups according to discharge strategy (Figure 1).

### 2.4. Allocation to One of the Strategies

The preferred strategy during the recruitment period was SDD. Patients who met at least one of the following criteria were allocated to the NDD strategy:

Patients admitted to other hospitals who were referred solely for ablation and returned to the referring hospital after the 8 h monitoring period;

Patients lacking adequate care and support at home during the first 48 h;

CAP completed after 13:00, precluding at least 8 h of monitoring before discharge;

Patients admitted for other reasons who did not meet the discharge criteria because of an extracardiac illness.

### 2.5. Same-Day Discharge Strategy Description

The SDD strategy followed essentially the same direction as NDD with the exception of the prolonged compresion bandaging time, which was shortened to 8 h after catheter removal. Regarding anticoagulation mananging, all patients followed our protocol, consisting of the interruption and resumption of anticoagulation in the morning and in the evening of the procedure, respectively, in case of DOAC use, and the continuation without any interruption in case of anti-VitK. Intraprocedural heparin aimed for ACT between 300 and 350, and protamine was not routinely used. With regard to vascular-access managing, most of the patients received 3 femoral venous puctures and haemostasis was accomplished with femoral sutures. Vascular ultrasound was not routinely used and depended on the operator. After 8 h of postprocedural monitoring, sutures were removed and patients initiated deambulation. Detailed instructions were given to the patient in case of any doubt or possible complication, together with a directions form, before discharge. Afterwards, follow-up was exactly the same for patients in NDD.

### 2.6. Study Variables, Definition of Complications, and Follow-Up

The medical records of patients allocated to SDD and NDD were reviewed and compared with respect to baseline demographic variables, procedural characteristics, outcomes (including complications), and detailed costs.

Minor and major complications were classified according to their timing: early complications occurred during CAP or within the first 8 h after CAP, whereas late complications occurred from 8 h after CAP through 6 months. However, patients who experienced major complications during CAP or within the first 8 h were not included in the analysis to avoid selection bias, as none of these patients would have been allocated to the SDD strategy.

Minor complications were defined as those that resolved spontaneously or with standard treatment, such as analgesics, antiemetics, or fluid therapy (e.g., reversible phrenic nerve palsy or urinary retention). Major complications were defined as those requiring more intensive measures (e.g., intensive fluid therapy, vascular suturing, surgery, vasoactive drugs, or pericardial drainage) or resulting in permanent phrenic nerve palsy. Significant complications were defined as events initially suggestive of a major complication that were not confirmed over the following hours but nevertheless resulted in exclusion from the final analysis in most of the cases.

The follow-up period was 6 months. Reports from scheduled visits and urgent medical encounters were reviewed to collect information on possible complications and outcomes. Only complications potentially related to CAP were considered in the final analyses.

### 2.7. Endpoints

Primary endpoint: The primary endpoint was a comparison of a composite safety endpoint, defined as any minor or major complication potentially related to CAP, between patients managed with the SDD and NDD strategies.

Secondary endpoints: Secondary endpoints included the identification of predictors of SDD. This analysis examined baseline demographic variables, procedural characteristics, and clinical outcomes as predictors of discharge strategy (SDD or NDD). A healthcare cost-analysis comparing the two discharge strategies was also performed. Differences in costs were primarily attributable to the overnight hospital stay associated with NDD. These costs included mandatory protocol-based testing (electrocardiography [ECG]), any additional tests required (blood tests or chest radiography), and the cost of a cardiology ward bed.

### 2.8. Statistical Analysis

Sample size: The sample size was calculated on the basis of published data on complications after CAP with an overnight stay, including minor complications. The planned sample comprised 135 and 270 patients in the SDD and NDD groups, respectively, to detect an expected difference between event rates of 0.08 in the first group and 0.17 in the second, with 70% power, an alpha level of 0.05, and a dropout rate of 10%.

Data analyses: Data are presented as mean ± standard deviation (SD), median and interquartile range [IQR], or number and proportion, according to variable type (continuous or categorical). Normality was assessed using the Shapiro–Wilk test. Differences in the distribution of categorical variables were evaluated using the Z-test. Continuous variables were compared using the Student *t*-test or Mann–Whitney U test, as appropriate. Multivariable binary logistic regression and comparative cost-analysis were subsequently performed. All statistical analyses were conducted using IBM SPSS Statistics version 28 (IBM Corp., Armonk, NY, USA), with 95% confidence intervals. Results were considered statistically significant at *p* ≤ 0.05.

## 3. Results

A total of 250 patients underwent CAP at our centre between 2021 and July 2024. Of these, 45 did not meet the eligibility criteria and were excluded from the analysis. Because the objective was to assess the safety of the SDD strategy, 33 patients who experienced significant complications during the ablation procedure (*n* = 5) or within the first 8 h of post-procedural monitoring (*n* = 28) were excluded to avoid selection bias. One patient who refused discharge and 11 medically complex patients with pre-existing conditions requiring prolonged admission unrelated to the ablation procedure were also excluded. The results presented below therefore correspond to the remaining 205 participants, who were assigned to either SDD or NDD (Figure 1).

### 3.1. Baseline Demographics

Patients in the SDD and NDD groups had similar baseline demographic and clinical characteristics (Table 1), including age, the presence of structural heart disease (SHD), arrhythmia type, medication use, and left ventricular ejection fraction (LVEF). Only residence outside the basic health zone differed, being more prevalent in the NDD group than in the SDD group (43.7% vs. 27.1%; *p* = 0.021).

### 3.2. Procedural Characteristics

Procedural details are presented in Table 2. ICE was used in all 205 patients (100%) as an important safety measure. Procedural success rates did not differ between strategies. Significant differences were observed in the time of CAP completion. CAP was completed by 13:00 in 75.7% of patients in the SDD group, compared with only 19.3% of those in the NDD group (*p* < 0.001). Use of the SDD strategy increased over time, from 14.6% in 2021 to 49.2% in 2023 (*p* < 0.001). The most common venous femoral access approach involved three punctures: two in the left lower extremity (92.2%) and one in the right (80.5%). The median numbers of total femoral access punctures and left femoral access punctures per patient were higher in the NDD group than in the SDD group (3 [IQR = 0] vs. 3 [0], *p* = 0.009; and 2 [0] vs. 2 [0], *p* = 0.012, respectively). No difference was observed in the number of right femoral access punctures. Median femoral compression bandage duration was longer in the NDD group than in the SDD group (18 [IQR = 4] vs. 8 [1] h, *p* < 0.001).

### 3.3. Complications

#### 3.3.1. Significant Early Complications (<8 h)

We observed 41 significant complications (16.4%) during CAP or within the first 8 h after CAP. These events resulted, in some cases, in exclusion from the final analysis to avoid selection bias. Eight (3.2%) were classified as major complications, whereas the remaining 33 (13.2%) were initially considered significant but were ultimately classified as minor complications (Table 3, total screened column). Of those 33 patients, 11 entered to final analysis due to a rapid identification of a minor complication judged to be not relevant (3 patients with an acute urinary retention, 2 patients with chest pain, 1 patient with palpitations, 1 patient with reversible phrenic nerve palsy, 2 patients with minor haematoma, and 2 patients with mild groin pain).

#### 3.3.2. Complications in the Final Analysis Group

Complications in the final analysis cohort (Table 3) were classified by severity as minor or major; mortality was also assessed. No significant differences were observed between the strategies. There were 11 early complications, all of which were minor: 6 (8.6%) in the SDD group and 5 (3.7%) in the NDD group (*p* = 0.191).

A total of 8 late complications occurred: 6 (4.4%) in the NDD group and 2 (2.9%) in the SDD group (*p* = 0.781). Minor complications, major complications, and deaths were numerically more frequent in the NDD group, although none of these differences were statistically significant (3.7% vs. 2.9%, *p* = 1; 0.7% vs. 0%, *p* = 1; and 3.7% vs. 0%, *p* = 0.168, respectively). Among all major complications, the only procedure-related life-threatening event was one case of cardiac tamponade requiring drainage. No procedure-related deaths occurred.

### 3.4. Costs

The incremental cost of the NDD strategy was €479.84, including the cardiology ward stay, meals, and additional testing (blood tests and ECG), with a further €248.09 for echocardiography and chest radiography when required. Between 2021 and July 2024, 70 patients underwent CAP and were discharged on the same day, resulting in an estimated cost reduction of at least €33,588.80 compared with NDD. Because no significant differences in complications or mortality were observed between strategies, these findings indicate that substantial savings were achieved without compromising safety. If SDD had been applied to all patients in the NDD group (*n* = 135), an additional €64,778.40 ± €33,492.15 could have been saved.

## 4. Discussion

The concept of an SDD strategy is not new. It has been used in cardiology since the late 20th century, initially after pacemaker implantation [6]. Early-discharge strategies are now used across a range of procedures, including transcatheter aortic valve replacement [7] and left atrial appendage closure [8,9]. Available prospective studies [5,8,10,11] have demonstrated these practices to be safe and feasible. These findings support their adoption to optimise resource use while maintaining patient safety.

We report a single-centre experience implementing an SDD strategy for CAP. Our findings suggest that SDD is both safe and cost-saving. Previous evidence on SDD after ablation is almost exclusively limited to atrial fibrillation (AF) [5,8,12], and few studies have implemented this strategy for other complex arrhythmia substrates. For example, patients undergoing CAP for ventricular arrhythmias (VAs) have generally been monitored for at least 1 day [13]. At our centre, however, SDD has been successfully implemented after procedures including VA ablation, cardioneuroablation, ablation of left-sided accessory pathways, and ablation of substrates other than AF, although this arrhythmia was the most treated for this study.

### 4.1. Complications with SDD Strategy

Previous reports [12] have shown that 8% of patients were ineligible for SDD because of major complications. In our study, 3.2% of patients experienced major complications as defined, although only one was life-threatening, and 13.2% were excluded from the final analysis because of significant complications. This approach should be interpreted in light of the fact that some patients whose events ultimately proved to be minor were excluded to avoid selection bias. We recorded a higher total number of minor complications than previous studies [12]. Given the nature of the present study, we included all events, including several that are not routinely recorded in other registries (e.g., acute urinary retention, palpitations, minor haematoma, and chest pain), thereby increasing the number of trivial complications. Furthermore, definitions of early versus late and minor versus major complications, as well as variable definitions and follow-up periods, differ substantially between studies. Reported complication rates also vary considerably across substrates and studies [13,14,15,16,17], ranging from 1% to 17% for all catheter ablation complications. In our initial cohort, the overall complication rate, including trivial events occurring throughout follow-up, was 20.4%. This may reflect the specific variables recorded (e.g., minor hypotension and acute urinary retention) or the inclusion of CAPs for substrates other than AF (e.g., ventricular arrhythmias), an area requiring further investigation because data on SDD remain limited. Overall, our findings are consistent with previous studies [17,18] in showing no statistically significant differences between SDD and NDD in early or late complications or mortality.

### 4.2. Cost Reduction with SDD Strategy

This study showed a progressive annual increase in the number of CAPs performed from 2021 to 2024. This is consistent with the Spanish catheter ablation registry, which reported a marked increase after the COVID-19 pandemic followed by a plateau, with 23,360 procedures performed in 2022 [19] and 26,207 in 2023 [1]. This growth imposes substantial costs on the public healthcare system. Previous studies have reported significant cost savings with an SDD strategy [14,20], consistent with our results. We observed a clear cost benefit of €479.84 ± €248.09 per CAP with this approach. Provided that SDD does not compromise safety, it should be considered the preferred strategy whenever feasible. The use of ICE in all cases facilitated the immediate detection of potential complications, thereby reducing the risk of life-threatening events [21]. Although ICE increases procedural costs, it provides important safety benefits [22,23,24] and is useful for both preprocedural planning and assessment of ablation results. For these reasons, this increasingly used technology has already been adopted by several centres [22,25,26]. On the other hand, based on previous studies [27], appropriate counselling, clear written medication instructions, and caregiver involvement are essential to minimise post-discharge complications, which could otherwise increase the indirect healthcare costs associated with SDD.

### 4.3. Predictors for an SDD Strategy

This study was conducted at a referral centre for CAP. Consequently, a substantial proportion of patients lived far from the hospital and required an overnight stay. Only 13.5% of the 126 patients whose CAP ended after 13:00 were allocated to SDD, compared with 67.1% of the 79 patients whose CAP ended before 13:00. These findings may help optimise electrophysiology laboratory scheduling, support the development of new protocols [8], and achieve cost savings without compromising safety.

With regard to patient selection for SDD, our findings suggest that most variables do not determine the likelihood of receiving this strategy. Instead, the decision appears to be driven primarily by the time of CAP completion, which is directly associated with the duration of femoral compression bandaging because the bandage remains in place until the following morning in patients allocated to NDD. Nevertheless, larger prospective studies are needed to determine whether variables other than those identified here influence the likelihood of SDD.

### 4.4. Limitations

This was not a randomised clinical trial comparing SDD and NDD after CAP but a single-centre observational study with a moderate sample size and limited power to detect differences in the primary safety outcomes. Nevertheless, no significant differences in late minor or major complications or mortality were observed between the discharge strategies. We considered major and minor late complications as our primary endpoint, even considering the differences in nature and prognosis between them. The reason to consider all complications together was taken for two reasons. First, we expected a low number of major late complications, with almost all of them minor. Second, our aim was to consider all possible complications, in order to consider SDD as a valid strategy.

As an observational study, this analysis may be subject to selection bias, with patients at higher risk of complications being more likely to undergo NDD. Conversely, some patients may have developed CAP-related complications that were not captured because data collection was limited to visits and healthcare centres within a specific region of Spain. Moreover, because costs were derived from a Spanish centre, the findings may not be generalisable to other healthcare systems. ICE was used in all patients, although its use is not widespread in Spain; consequently, our results may be most applicable to centres that routinely use ICE for CAP. Similarly, AF was the predominant arrhythmic substrate in our cohort, which may limit generalisability to other CAP substrates.

Most patients in our study underwent Z-suture closure after CAP, whereas a ProGlide vascular closure system was used in only two cases. More extensive use of ProGlide or another vascular closure system might have reduced bleeding complications; however, our findings cannot be extrapolated to patients managed with these haemostatic devices. Future research should therefore assess complications associated with these devices in the context of SDD. Although the existing literature is limited, it suggests promising outcomes [18] and a potential additional effect on cost savings.

## 5. Conclusions

An SDD strategy following CAP for AF might be safe, and it is feasible and cost-saving. Further prospective, randomised studies are needed to comprehensively assess the safety of SDD after CAP across a broader range of arrhythmic substrates and to confirm the potential cost reductions.

**What is known about the topic?**

The COVID-19 pandemic limited access to healthcare, leading to the adoption of same-day discharge strategies that could potentially compromise patient safety.

Expanded indications for complex arrhythmias have increased the number and scope of complex ablation procedures. This growing demand, combined with limited resources, presents a new challenge for which early-discharge strategies may be necessary.

**What does this study add?**

In complex ablation procedures, which often require access to the left heart chambers, safety remains a priority. Relevant measures include the use of intracardiac echocardiography and femoral suturing after ablation. A same-day discharge strategy did not adversely affect acute procedural success, complication rates, or mortality.

This feasible, cost-saving same-day discharge strategy can be applied to atrial fibrillation procedures and maybe to other complex ablation procedures.

## Figures and Tables

**Figure 1:**
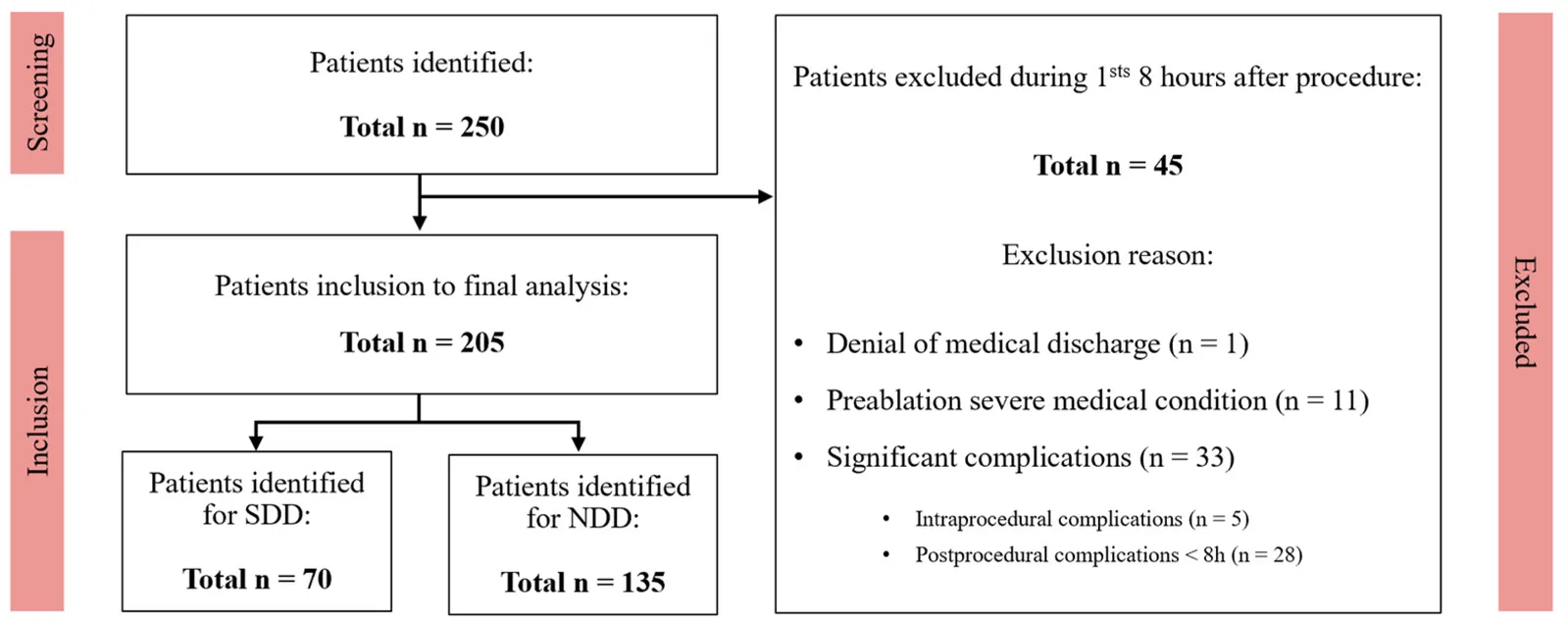
Flow chart of patients included in the study. NDD: next-day discharge, SDD: same-day discharge.

**Table 1:** Comparison of baseline demographics: SDD vs. NDD.

	Total *n* = 205	SDD *n* = 70	NDD *n* = 135	*p*-Value
Outside basic health zone, ***n*** (%)	78 (38)	19 (27.1)	59 (43.7)	**0.021**
Age, average ± standard deviation	60.12 [14.30]	58.97 [17.44]	60.77 [10.35]	0.365
Female, ***n*** (%)	57 (27.8)	24 (34.3)	33 (24.4)	0.136
BMI, average ± standard deviation	27.72 [5.93]	28.15 [5.84]	27.45 [5.76]	0.267
LA diameter, average ± standard deviation	40 [8]	40 [8]	40 [8]	0.584
LA dilatation (≥40 mm), ***n*** (%)	113 (55.7)	36 (52.9)	77 (57)	0.579
LVEF (%), average ± standard deviation	60 [11]	60 [10]	61 [12]	0.736
LVEF reduced function (<50%), ***n*** (%)	34 (17.3)	9 (13.4)	25 (19.2)	0.308
HBP, ***n*** (%)	120 (58.5)	41 (58.6)	79 (58.5)	0.994
DM, ***n*** (%)	44 (21.5)	15 (21.4)	29 (21.5)	0.993
DLP, ***n*** (%)	76 (37.1)	25 (35.7)	51 (37.8)	0.772
GFR (≤60 mL/min/1.73 m^2^), ***n*** (%)	31 (15.1)	13 (18.6)	18 (13.3)	0.321
IHD, ***n*** (%)	24 (11.7)	7 (10)	17 (12.6)	0.584
SHD, ***n*** (%)	62 (30.2)	19 (27.1)	43 (31.9)	0.486
Hypertrophic cardiomyopathy	5 (2.4)	3 (4.3)	2 (1.5)	
Dilated cardiomyopathy	10 (4.9)	2 (2.9)	8 (5.9)	
Arrhythmogenic cardiomyopathy	2 (1)	1 (1.4)	1 (0.7)	
Valvular heart disease	35 (17.1)	9 (12.9)	26 (19.3)	
Ischemic dilated cardiomyopathy	4 (2)	2 (2.9)	2 (1.5)	
Others	6 (2.9)	2 (2.9)	4 (3)	
Arrhythmia type, ***n*** (%)				0.162
Atrial fibrillation	151 (73.7)	52 (74.3)	99 (73.3)	
Atypical atrial flutter	19 (9.3)	3 (4.3)	16 (11.9)	
Ventricular arrhythmia	13 (6.3)	7 (10)	6 (4.4)	
Others	22 (10.7)	8 (11.4)	14 (10.4)	
Previous ablation, ***n*** (%)	71 (34.6)	25 (35.7)	46 (64.8)	0.877
Anticoagulation, ***n*** (%)	172 (83.9)	56 (80)	116 (85.9)	0.274
Antiplatelets, ***n*** (%)	7 (3.4)	2 (2.9)	5 (3.7)	1

BMI: body mass index, DM: diabetes mellitus, DLP: dyslipidaemia, GFR: glomerular filtration rate, HBP: high blood pressure, IHD: ischemic heart disease, LA: left atrial, LVEF: left ventricular ejection fraction, NDD: next-day discharge, SHD: structural heart disease, SDD: same-day discharge, VA: ventricular arrhythmia. Values represent number (%), or median [interquartile range].

**Table 2:** Procedural characteristics comparing SDD vs. NDD.

	Total *n* = 205	SDD *n* = 70	NDD *n* = 135	*p*-Value
Annual complex ablation count, ***n*** (%)				**<0.001**
2021	48 (23.4)	7 (10)	41 (30.4)	
2022	58 (28.3)	16 (22.9)	42 (31.1)	
2023	65 (31.7)	32 (45.7)	33 (24.4)	
2024 (first semester)	34 (16.6)	15 (21.4)	19 (14.1)	
Ablation technology, ***n*** (%)				0.210
RF ablation	132 (64.4)	41 (58.6)	91 (67.4)	
CB ablation	73 (35.6)	29 (41.4)	44 (32.6)	
Acute procedure success, ***n*** (%)				0.252
Failure	3 (1.5)	0 (0)	3 (2.2)	
Partial	17 (8.3)	5 (7.1)	12 (8.9)	
Complete	185 (90.2)	65 (92.9)	120 (88.9)	
Anaesthesia, ***n*** (%)				0.218
Conscious sedation	130 (63.4)	47 (67.1)	83 (61.5)	
Deep sedation	29 (14.1)	12 (17.1)	17 (12.6)	
General anaesthesia	46 (22.4)	11 (15.7)	35 (25.9)	
ICE, ***n*** (%)	205 (100)	70 (100)	135 (100)	
Left approach, ***n*** (%)				0.470
Transeptal	204 (99.5)	70 (100)	134 (99.3)	
Retroaortic	1 (0.5)	0 (0)	1 (0.7)	
Femoral access number, ***n*** (%)				**0.009**
2	7 (3.4)	5 (7.1)	2 (1.5)	
3	162 (79)	58 (82.9)	104 (77)	
4	36 (17.6)	7 (10)	29 (21.5)	
Number of left femoral access, ***n*** (%)				**0.012**
0	4 (2)	3 (4.3)	1 (0.7)	
1	12 (5.9)	8 (11.4)	4 (3)	
2	189 (92.2)	59 (84.3)	130 (96.3)	
Number of right femoral access, ***n*** (%)				0.118
1	165 (80.5)	58 (82.9)	107 (79.3)	
2	36 (17.6)	9 (12.9)	27 (20)	
3	4 (2)	3 (4.3)	1 (0.7)	
Maximum sheath size, ***n*** (%)				0.316
≤13 Fr	54 (26.3)	15 (21.4)	39 (28.9)	
>13 Fr	151 (73.7)	55 (78.6)	96 (71.1)	
Femoral artery (patients)	5 (2.4)	2 (2.9)	3 (2.2)	0.780
Preprocedural imaging, ***n*** (%)				
TTE or TEE	194 (96)	68 (98.6)	126 (94.7)	0.269
CCT	16 (7.9)	6 (8.7)	10 (7.5)	0.769
CMR	15 (7.4)	5 (7.2)	10 (7.5)	0.944
Femoral suture (Z or 8), ***n*** (%)	197 (96.1)	65 (92.9)	132 (97.8)	0.125
Arterial proglide, ***n*** (%)	2 (1)	0 (0)	2 (1.5)	0.548
End time of CAP before 13:00, ***n*** (%)	79 (38.5)	53 (75.7)	26 (19.3)	**<0.001**
Compression bandage time (hours), ***n*** (%)	16 [11]	8 [1]	18 [4]	**<0.001**

CAP: complex ablation procedure, CB: cryoballoon, CCT: cardiac computed tomography, CMR: cardiac magnetic resonance, Fr: French size, ICE: Intracardiac echocardiography, NDD: next-day discharge, RF: radiofrequency, SDD: same-day discharge, TEE: transoesophageal echocardiography, TTE: transthoracic echocardiography. Values represent number (%) or median [interquartile range].

**Table 3:** Complications, including minor and major complications, and mortality. Each patient could contribute to more than one complication, but this circumstance did not happen.

	Total Screened *n* = 250	Total Included *n* = 205	SDD *n* = 70	NDD *n* = 135	*p*-Value (SDD vs. NDD)
Minor < 8 h, ***n*** (%)	33 (13.2)	11 (5.4)	6 (8.6)	5 (3.7)	0.191
AUR	4 (1.6)	3 (1.5)	2 (2.9)	1 (0.7)	0.102
Chest pain	6 (2.4)	2 (1)	0 (0)	2 (1.5)	
Palpitations	2 (0.8)	1 (0.5)	1 (1.4)	0 (0)	
RPNP	9 (3.6)	1 (0.5)	0 (0)	1 (0.7)	
Haematoma/bleed AS	7 (2.8)	2 (1)	1 (1.4)	1 (0.7)	
Pericarditis	1 (0.4)	0 (0)	0 (0)	0 (0)	
Hypotension	2 (0.8)	0 (0)	0 (0)	0 (0)	
Others	2 (0.8)	2 (1)	2 (2.9)	0 (0)	
Major < 8 h, ***n*** (%)	8 (3.2)	NA	NA	NA	NA
PNP	2 (0.8)				
Vascular injury	1 (0.4)				
PLE (no drain)	1 (0.4)				
PCE (no drain)	2 (0.8)				
Tamponade (surgery)	1 (0.4)				
Heart failure	1 (0.4)				
Minor 8 h-6 m, ***n*** (%)	9 (3.6)	7 (3.4)	2 (2.9)	5 (3.7)	1
AUR	1 (0.4)	1 (0.5)	0 (0)	1 (0.7)	0.840
Haematoma/bleed AS	5 (2)	3 (1.5)	1 (1.4)	2 (1.5)	
Pericarditis	3 (1.2)	3 (1.5)	1 (1.4)	2 (1.5)	
Major 8 h-6 m, ***n*** (%)	1 (0.4)	1 (0.5)	0 (0)	1 (0.7)	1
Haematoma/bleed AS	1 (0.4)	1 (0.5)	0 (0)	1 (0.7)	1
All complications 8 h-6 m	10 (4)	8 (3.9)	2 (2.9)	6 (4.4)	0.781
All complications, ***n*** (%)	51 (20.4)	18 (8.8)	8 (11.4)	10 (7.5)	0.351
Mortality at 6 m, ***n*** (%)					
Death	7 (2.8)	5 (2.4)	0 (0)	5 (3.7)	0.168
Related to procedure	0 (0)	0 (0)	0 (0)	0 (0)	

AS: access site, AUR: acute urinary retention, h: hour, m: months, NA: not applicable, NDD: next-day discharge, PCE: pericardial effusion, PLE: pleural effusion, PNP: phrenic nerve palsy, RPNP: reversible phrenic nerve palsy, SDD: same-day discharge. Values represent number (%).

## Data Availability

The datasets used and/or analysed during the current study are available from the corresponding author on reasonable request.

## Abbreviations

CAP: complex ablation procedure. ICE: intracardiac echocardiography. LVEF: left ventricle ejection fraction. NDD: next-day discharge. SDD: same-day discharge. SHD: structural heart disease.
